# Pathophysiological Roles of Actin-Binding Scaffold Protein, Ezrin

**DOI:** 10.3390/ijms23063246

**Published:** 2022-03-17

**Authors:** Kotoku Kawaguchi, Shinji Asano

**Affiliations:** Department of Molecular Physiology, College of Pharmaceutical Sciences, Ritsumeikan University, 1-1-1 Noji-Higashi, Kusatsu 525-8577, Japan; k-kawagu@fc.ritsumei.ac.jp

**Keywords:** ezrin, cytoskeleton, actin, G protein, cancer, metastasis, ciliogenesis

## Abstract

Ezrin is one of the members of the ezrin/radixin/moesin (ERM) family of proteins. It was originally discovered as an actin-binding protein in the microvilli structure about forty years ago. Since then, it has been revealed as a key protein with functions in a variety of fields including cell migration, survival, and signal transduction, as well as functioning as a structural component. Ezrin acts as a cross-linker of membrane proteins or phospholipids in the plasma membrane and the actin cytoskeleton. It also functions as a platform for signaling molecules at the cell surface. Moreover, ezrin is regarded as an important target protein in cancer diagnosis and therapy because it is a key protein involved in cancer progression and metastasis, and its high expression is linked to poor survival in many cancers. Small molecule inhibitors of ezrin have been developed and investigated as candidate molecules that suppress cancer metastasis. Here, we wish to comprehensively review the roles of ezrin from the pathophysiological points of view.

The ezrin/radixin/moesin (ERM) proteins are general cross-linkers between cortical actin filaments and the plasma membrane. They are concentrated at cell surface structures such as microvilli, filopodia, ruffling membranes, uropods, retraction fibers, and cell adhesion sites, where actin filaments are associated with plasma membranes. The ERM proteins consist of an N-terminal FERM domain (named after the 4.1 protein, ezrin, radixin, and moesin) and a C-terminal domain containing an F-actin binding site. These two domains are connected by a central α-helix structure (Figure 1). The FERM domain, which is composed of three structural modules (F1, F2 and F3), forms a compact clover-shaped structure [1] and is involved in the binding of membrane proteins, as well as scaffold proteins as reviewed previously [2,3,4].

Ezrin is one of the ERM family proteins. The function of ezrin is regulated by the interaction between its N- and C-terminal domains. This interaction masks the binding sites for membrane proteins located in the FERM domain, and the binding site for actin filaments located in the C-terminal domain, which makes ezrin dormant in its closed conformation (Figure 2) [2,4,5]. This property seems to be common among all ERM proteins. The closed form of ezrin resides in the cytosolic fraction of cells. The binding of phosphatidylinositol 4,5-bisphosphate (PtdIns(4,5)P_2_) to the FERM domain (known as priming) [4,6] and the subsequent phosphorylation of a threonine residue (Thr^567^) located in the C-terminal domain by protein kinases such as lymphocyte-oriented kinase (LOK), STE20-like protein kinase (SLK), and Rho kinase open the closed conformation into the active conformation [7,8,9]. In these activation processes, ezrin is recruited from the cytoplasm (in a soluble inactive form) to the membrane (in an insoluble active form), and oscillates between the open and closed conformations by cycles of phosphorylation and dephosphorylation [8].

Here, we wish to introduce ezrin as a multifunctional protein. (1) Ezrin is a general cross-linker which binds membrane proteins such as receptors, ion channels, and transporters to the actin cytoskeleton directly or indirectly through scaffold proteins. It also connects the extracellular matrix to the actin cytoskeleton. (2) Ezrin functions as a regulator of G-protein-related proteins. (3) Ezrin acts as a scaffold protein which recruits proteins involved in signal transduction. (4) Ezrin anchors cyclic AMP-dependent protein kinases (A-kinases). (5) Ezrin is involved in the generation of cilia: ciliogenesis. (6) Ezrin is closely linked with the metastatic progression of cancer. In fact, its high expression is linked to poor survival in many cancers. (7) Ezrin regulates mRNA translation as one of the components of the ribonucleoprotein complex.

We wish to introduce these points in detail in the following sections.

## 1. The Role of Ezrin as the Cross-Linker between Membrane Proteins and the Actin Cytoskeleton

**Figure 3:**
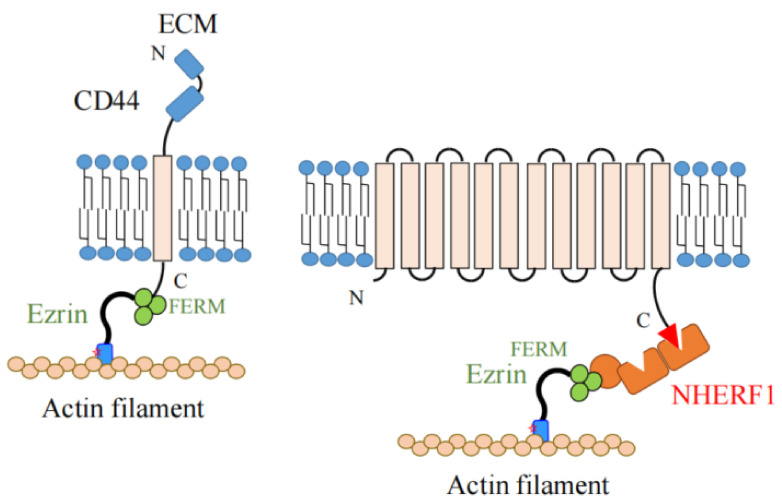
Ezrin functions as a cross-linker between membrane proteins and actin filaments directly or indirectly via scaffold proteins. (**Left**) Ezrin directly cross-links cell adhesion molecules such as CD44 and actin filaments. In this case, ezrin plays a key role in intermolecular communication between adhesion proteins attached to the extracellular matrix (ECM) and the actin cytoskeleton. (**Right**) Ezrin cross-links ion channels (such as CFTR), transporters (such as NHE3, Npt2a), and receptors (such as β_2_AR) to actin filaments indirectly via scaffold proteins such as NHERF1 (shown by orange boxes and circle). In this case, ezrin binds to the ERM binding site (shown by an orange circle) of scaffold proteins via the FERM domain (shown by clover-shaped green circles).

Ezrin is a protein which links membrane proteins such as receptors, ion channels, and transporters to the actin cytoskeleton directly or indirectly through scaffold proteins, as shown in Figure 3.

### 1.1. Direct Interactions with Membrane Proteins

Proteins which directly bind to ezrin (or ERM proteins) are summarized, alongside their functional roles, in Table 1.

Ezrin binds to cell adhesion proteins with a single transmembrane segment such as CD44 (the receptor for hyaluronic acid), CD43, intercellular adhesion molecule (ICAM)-1 and 2, and L-selectin at the juxtamembrane amino acid sequences in their cytoplasmic domains [10,11,12]. In macrophages, ezrin tethers CD44 to the actin cytoskeleton, and CD44 functions as a picket to limit the diffusion of the Fc receptor in the plasma membrane [13]. L-selectin is expressed in leukocytes and binds to ezrin in its resting state and during early transendothelial migration. This interaction is involved in the recruitment of monocytes to activated endothelial monolayers [14]. Therefore, ezrin plays a key role in the intermolecular communication between adhesion proteins and the actin cytoskeleton.

Ezrin is also involved in the regulation of spatiotemporal organization and the formation of nanoclusters of membrane proteins [15]. Ezrin directly binds to several transporters to regulate their surface expression and endocytosis. The protein Na^+^, H^+^-exchanger 1 (NHE1) contains a positively-charged ERM-binding motif in the C-terminal cytoplasmic tail, which binds to the FERM domain of ezrin [16]. The ERM proteins, especially ezrin, also directly bind to the P-glycoprotein (P-gp), which is a key player in multidrug-resistant phenotypes in cancer. Ezrin increases the cell surface expression and transport functions of P-gp [17]. Therefore, ezrin seems to play a key role in driving the multidrug resistance of tumors [18]. Ezrin also directly binds to ATP11C-b, which translocates membrane phospholipids as a flippase, at the FERM domain. ATP11C-b contains the quadruplet LLXY amino acid sequence as a binding motif in its C-terminal cytoplasmic tail. Ezrin is not directly involved in flippase activity but is important for stable polarized expression of ATP11C-b [19].

Ezrin also binds to G-protein-related proteins and signaling molecules as described in the following sections.

### 1.2. Indirect Interactions with Membrane Proteins

Proteins which indirectly bind to ezrin (or ERM proteins) are summarized, alongside their functional roles, in Table 1.

Ezrin functions as a cross-linker between membrane proteins and the actin cytoskeleton indirectly through scaffold proteins. The scaffold proteins Na^+^/H^+^ exchanger regulatory factors (NHERF) 1 and 2, which contain two PDZ (PSD-95, Discs-large, and ZO-1) domains, bind to the FERM domain of ezrin at their C-termini [20,21]. These PDZ domains on the NHERFs interact with the PDZ-binding motifs (located at the C-termini) of membrane proteins such as the cystic fibrosis transmembrane conductance regulator (CFTR) [22], Na^+^, H^+^-exchanger 3 (NHE3) [23], Na^+^-dependent phosphate transporter 2a (Npt2a) [24], and the β_2_-adrenergic receptor (β_2_AR) [25]. Consequently, multi-protein complexes linked to the actin cytoskeleton are formed close to the membrane, which is important for the cell surface expression and functional integration of membrane proteins.

### 1.3. The Role of Ezrin as a Regulator of Plasma Membrane Tension

**Figure 4:**
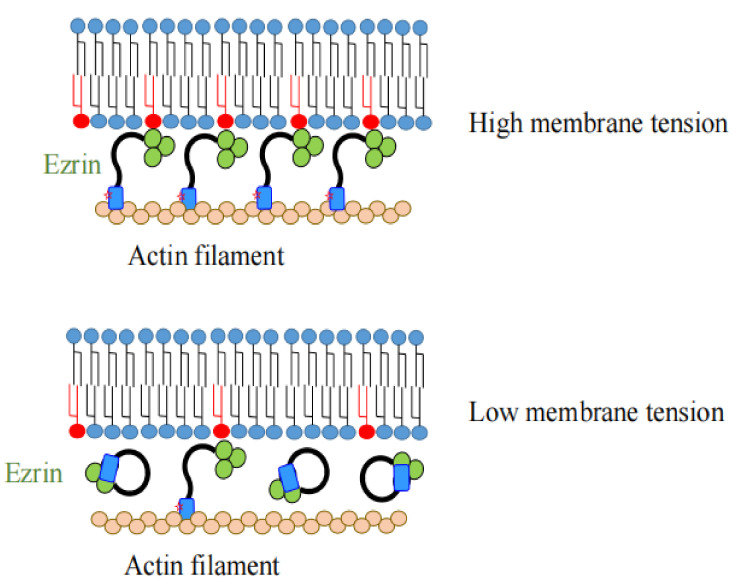
Ezrin functions as a regulator of plasma membrane tension. Plasma membrane tension is dominated by the attachment of actin filaments to the inner leaflet of the plasma membrane. Ezrin plays an important role by linking PtdIns(4,5)P_2_ (shown in red) located in the inner leaflet of the plasma membrane and the actin cytoskeleton. The membrane tension is increased by the increased amounts of PtdIns(4,5)P_2_ and by the activation of ezrin (shown in the upper part of figure). Conversely, it is decreased by the decreased amounts of PtdIns(4,5)P_2_ and by the inactivation of ezrin (shown in the lower part of figure).

Plasma membrane tension, which is responsible for many cellular functions such as cell migration, endocytosis, differentiation, and osmoregulation is dominated by the attachment of the actin cortex to the inner leaflet of the plasma membrane [26,27]. It is regulated by the ERM proteins, especially ezrin, which are the cross-linkers between the plasma membrane and the actin cytoskeleton. The membrane tension is increased by the addition of PtdIns(4,5)P_2_ and by the activation of ezrin, whereas it is decreased by the decreases in PtdIns(4,5)P_2_ and by the inactivation of ezrin via treatment with ezrin siRNA or a small molecule inhibitor of ezrin (Figure 4) [27]. A decrease in membrane tension is also found during the early differentiation of embryonic stem cells, which is initiated by a β-catenin-mediated decrease in the phosphorylation of ERM proteins [28].

## 2. The Role of Ezrin as the Regulators of Rho-Related Proteins

Ezrin integrates Rho-GTPase signaling and functions as both an upstream and downstream effector of Rho GTPases to regulate cytoskeletal organization [29]. Ezrin is activated by phosphorylation by Rho kinases, whereas it binds to Rho-related proteins to regulate Rho GTPase activity.

### 2.1. Interactions with Rho Guanine Nucleotide Exchange Factors (Rho-GEFs)

Rho-GEFs catalyze the exchange of GDP for GTP combined with Rho GTPases, and promote their activation. Here, we introduce two examples of such Rho-GEFs which interact with ezrin.

**Figure 5:**
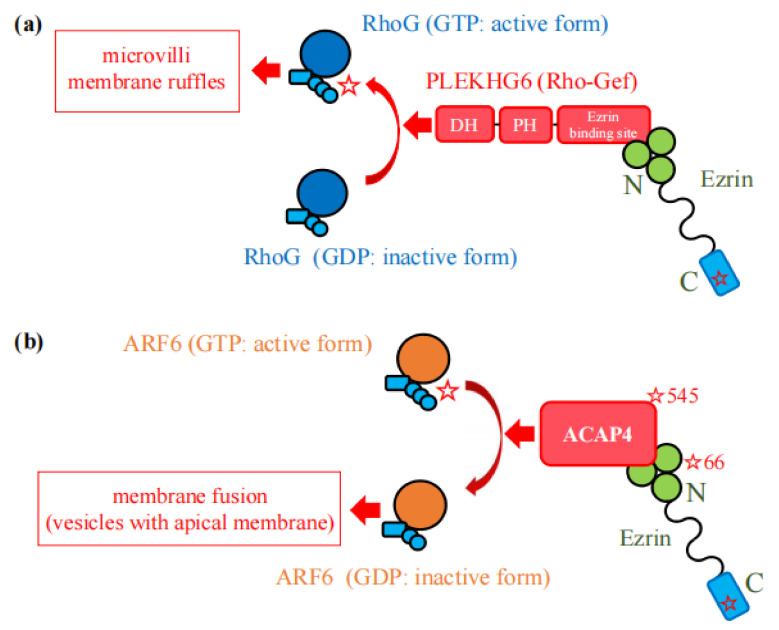
Ezrin activates Rho GTPases. (**a**) RhoG (shown by blue circles) is activated by ezrin/PLEKHG6 interactions. PLEKHG6 is shown by three tandem red boxes. DH and PH represent Dbl homology domain and plekstrin homology domain, respectively. The N-terminal FERM domain of ezrin (shown by a green clover) binds to the C-terminal region (ezrin binding site) of PLEKHG6. The Ezrin-PLEKHG6 complex activates its effector, RhoG, and finally induces the formation of microvilli and membrane ruffles. (**b**) ARF6 (shown by an orange circle) is activated by ezrin/ACAP4 interactions. ACAP4 is shown by a red box. Ezrin is phosphorylated at Ser^66^ and interacts with ACAP4 phosphorylated at Thr^545^. The Ezrin/ACAP4 complex and activated ARF6 together induce membrane fusion of intracellular tubulovesicles with the apical membrane.

Ezrin forms a complex with a Rho-GEF named “pleckstrin homology domain containing family G with RhoGef domain member 6” (PLEKHG6) via its N-terminal FERM domain (Figure 5a). Ezrin recruits PLEKHG6 and its effector, RhoG, to the apical pole of epithelial cells, where PLEKHG6 activates RhoG and induces the formation of microvilli and membrane ruffles and micropinocytosis, demonstrated in LLC-PK1 and A431 cells, respectively [30].

Another example of a Rho-GEF which interacts with ezrin is the engulfment and cell motility (ELMO) protein. ELMO interacts with DOCK1, a GEF of Rac1, and forms the ELMO-DOCK1 complex. Ezrin binds to this complex and activates Rac1 in human respiratory epithelial cells as shown in detail in Section 5 [31].

### 2.2. Interactions with GTPase-Activating Protein (GAP)

Ezrin phosphorylated at Ser^66^ by A-kinase functions as a scaffold for ACAP4 (especially ACAP4 phosphorylated at Thr^545^ by mammalian STE20-like protein kinase 4 (MST4)), which acts as a GTPase-activating protein (GAP) for ADP-ribosylation factor 6 (ARF6) [32,33]. ARF6 is a small GTPase which regulates membrane trafficking and requires the ability to cycle between active (GTP-bound) and inactive (GDP-bound) states for its function (Figure 5b). Ezrin, ACAP4, and cycling ARF6 are all necessary for the membrane fusion of intracellular vesicles with apical membranes and the proper localization of the gastric proton pump for gastric acid secretion. In fact, ezrin knockdown mice show achlorhydria and concomitant structural changes of the gastric glandular epithelia due to the impairment of membrane fusion [34,35].

### 2.3. Interactions with Rho GDP-Dissociation Inhibitors (Rho-GDIs)

Rho-GDIs are inhibitory regulators of all Rho family members, and interact to stabilize the inactive GDP-bound Rho GTPases. Ezrin interacts with Rho-GDI at the FERM domain. This interaction rescues the GDP-bound Rho GTPase from the Rho-GDP/GDI-complex, and activates Rho GTPase [36]. Here, we introduce an example of these effects on Rho-GDI in glomerular podocytes.

Podocytes have characteristic long foot processes, and are involved in filtration in Bowman’s capsules. Podocalyxin (PC) is a major sialoglycoprotein in podocytes, and regulates foot processes through the activation of a Rho family member, RhoA. Ezrin is associated with PC, and recruits Rho-GDI from the Rho-GDP/GDI-complex to activate RhoA and actin reorganization [37].

### 2.4. Functional Regulation of Ezrin by Rho-Associated Coiled-Coil Containing Kinase (ROCK)

ROCK is a downstream effector of RhoA [38,39], and directly phosphorylates myosin light chains, inhibits myosin phosphatase, and induces the assembly of stress fibers [40]. ROCK also phosphorylates ezrin at Thr^567^. Y27632, a ROCK inhibitor, blocks ezrin phosphorylation and binding to the actin cytoskeleton [41].

## 3. The Role of Ezrin as a Scaffold Protein-Recruiting Proteins Involved in Signal Transduction

Ezrin functions as a scaffold protein which recruits proteins involved in signal transduction, and determines the survival of epithelial cells. The epidermal growth factor (EGF) and hepatocyte growth factor (HGF) are major effectors that lead to the phosphorylation and activation of ezrin.

### 3.1. EGF-EGFR Signaling

**Figure 6:**
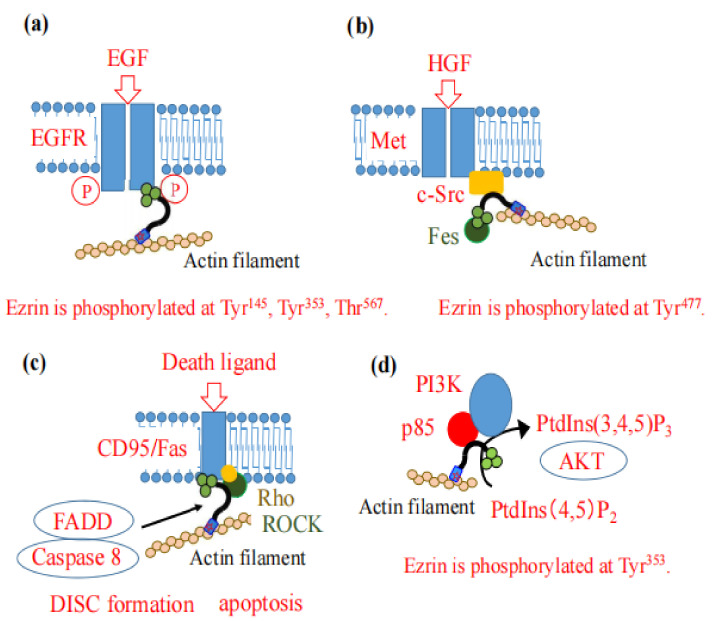
Ezrin is involved in several patterns of signal transduction. (**a**) In EGF signaling, ezrin cross-links the EGFR (shown by a couple of blue boxes) and actin filaments. In this process, ezrin itself is phosphorylated and activated by EGFR and other kinase(s) at Tyr^145^, Tyr^353^ and Thr^567^. (**b**) In HGF signaling, HGF binds its receptor tyrosine kinase, Met (shown by a couple of blue boxes), which phosphorylates a non-receptor tyrosine kinase, c-Src (shown by a yellow box). Ezrin is phosphorylated by c-Src at Tyr^477^, and interacts with Fes tyrosine kinase (shown by a green circle). Thus, Fes is indirectly cross-linked with the actin cytoskeleton, recruited at the sites of cell-cell contact, and functions in cell invasion and metastasis. (**c**) Ezrin cross-links the receptor of death ligand, CD95/Fas (shown by a blue box), and the actin cytoskeleton, which is followed by the association of FADD and caspase 8 to form the DISC complex for apoptosis. Rho (shown by a yellow circle) and ROCK (shown by a green box) also associate with the complex. (**d**) Ezrin binds to the regulatory subunit of PI3-kinase, p85 (shown by a red circle). PI3-kinase synthesizes PtdIns(3,4,5)P_3_, which activates its downstream target, protein kinase AKT, and protects against apoptosis.

Ezrin is phosphorylated at Tyr^145^ (in the FERM domain) and Tyr^353^ (in the central α-helix structure) in human A431 epidermoid carcinoma cells treated with EGF [42]. These phosphorylations are associated with the formation of microvillar-like surface structures [43], suggesting that ezrin interacts with the EGF receptor and promotes cellular differentiation. Recently, Saygideger-Kont, et al. reported the interaction between ezrin and EGFR in the same protein complex by immunoprecipitation, with their interaction being increased by treatment with EGF in human non-small cell lung cancer (NSCLC) cells [44]. They also reported that the phosphorylation of ezrin enhanced the interaction between ezrin and the EGFR. These results suggest that ezrin functions as a scaffold protein of the EGFR (Figure 6a).

### 3.2. HGF-Met Signaling

Ezrin also plays a major role in HGF/Met signaling-induced morphogenic effects. It is well-known that HGF interacts with its receptor Met, which possesses tyrosine kinase activity [45]. One of the proteins recruited by the phosphorylated c-Met is a non-receptor tyrosine kinase c-Src, which consequently phosphorylates ezrin at Tyr^477^. Finally, ezrin phosphorylated in this way directly interacts with Fes tyrosine kinase (Figure 6b). The interaction between Fes and ezrin at cell-cell contacts plays a major role in HGF-mediated cell scattering, local invasion, and metastasis [46]. In fact, the Y477F ezrin mutant reduced the number of lung metastasis events of breast carcinomas [47]. It was also reported that HGF/Met signaling activates the transcriptional factor specificity protein 1 (Sp1) through the MAPK pathway, and that activated Sp1 can in turn directly bind to the promotor of the ezrin gene and regulate its transcription in melanomas [48].

### 3.3. CD95 (APO-1/Fas)-Mediated Apoptosis

Ezrin is also involved in CD95 (APO-1/Fas)-mediated apoptosis. CD95, a member of the death receptor family, recruits the adaptor molecule Fas-associated death domain (FADD) protein and caspase 8, and forms the death-inducing signaling complex (DISC) to activate the apoptotic cascade [49]. Ezrin binds with CD95 at the FERM domain (amino acids 149–168) and cross-links CD95 and the actin cytoskeleton [50], which is an important initial event leading to DISC formation (Figure 6c) [49,51,52]. During the course of CD95 stimulation in Jurkat cells, ezrin is phosphorylated at Thr^567^ by ROCK in a Rho-dependent manner [53].

### 3.4. PI3-Kinase-AKT Signaling

Ezrin functions as a scaffold protein in the phosphatidylinositol 3-kinase (PI3-kinase) Akt pathway. Ezrin interacts with the regulatory subunit of PI3-kinase, p85 at the N-terminal FERM domain, and the phosphorylated Tyr^353^ (Figure 6d). The PI3-kinase activates its downstream target, protein kinase AKT, and protects cells against apoptosis [54].

## 4. The Role of Ezrin as the Protein Kinase A (PKA)-Anchoring Protein (AKAP)

**Figure 7:**
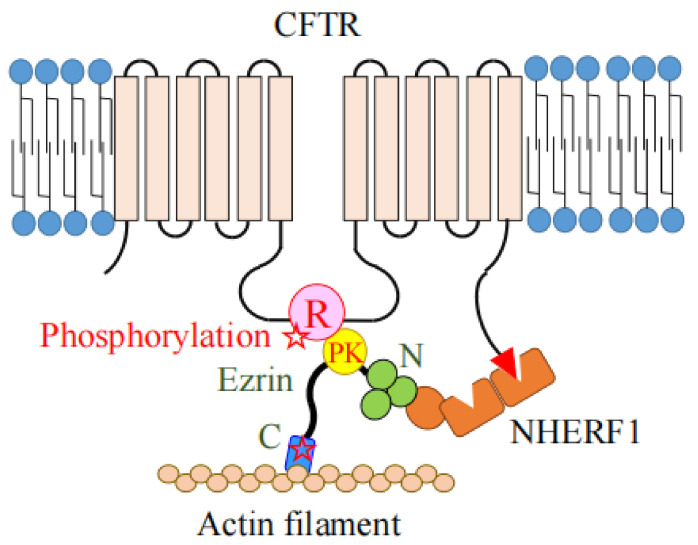
Ezrin functions as an AKAP for the efficient phosphorylation of CFTR. The function of CFTR is regulated by phosphorylation of its regulatory domain (shown by a pink circle with a letter of R) by protein kinase A (PKA) (shown by a yellow circle with a letter of PK). Ezrin simultaneously binds to a regulatory subunit of PKA directly, and CFTR via a scaffold protein, NHERF1 (shown by orange boxes and circle). This CFTR-AKAP (ezrin)-PKA association on the membrane provides the necessary specificity for the phosphorylation-dependent regulation of the CFTR channel.

Ezrin functions as a protein-kinase-A-anchoring protein (AKAP) which recruits the regulatory subunit of PKA into proximity with its substrates for their effective phosphorylation [55]. Ezrin binds to a type II A-kinase regulatory subunit R_II_ at the α−helix structure between the FERM domain and actin-binding domain (between amino acids 373 and 439) [55]. Ezrin binds to CFTR or NHE3 via the scaffold protein NHERF1 (EBP50) at the N-terminal FERM domain, on the other hand, it binds to the A-kinase regulatory subunit as an AKAP (Figure 7). CFTR is activated by cAMP-dependent phosphorylation at the regulatory (R) domain. Therefore, the CFTR-AKAP (ezrin)-PKA association can provide the necessary specificity for the phosphorylation-dependent regulation of CFTR channel gating [56]. Therefore, ezrin plays an important role in sequestering PKA close to its target proteins, CFTR and NHE3, for their effective phosphorylation [23,56].

Similar roles of ezrin as an AKAP have been reported in several studies. One such study observed the cAMP-dependent activation of gap junctions. Ezrin functions as an AKAP, and recruits PKA into close proximity with connexin 43, which enables its phosphorylation by PKA and the opening of gap junctions in cytotrophoblasts and liver epithelial cell lines [57,58].

Ezrin also functions as an AKAP to mediate T cell repression by cAMP in the negative signal pathway. In this process, ezrin recruits PKA Type I close to the C-terminal Src kinase (Csk) to promote its phosphorylation. In this way, the phosphorylated Csk in turn phosphorylates and inactivates lymphocyte-specific protein tyrosine kinase (Lck), which is a downstream component of T cell receptor signaling [59,60].

ERM proteins (including ezrin) function as an AKAP to mediate axon guidance in neuroblastomas in the netrin and its receptors, which are deleted in colorectal cancer (DCC) signaling. In this process, ERM proteins recruit PKA close to DCC, which is required for proper netrin/DCC-mediated signaling in growth cone morphology and neurite outgrowth [61].

## 5. The Role of Ezrin in Generation of Cilia, Ciliogenesis

**Figure 8:**
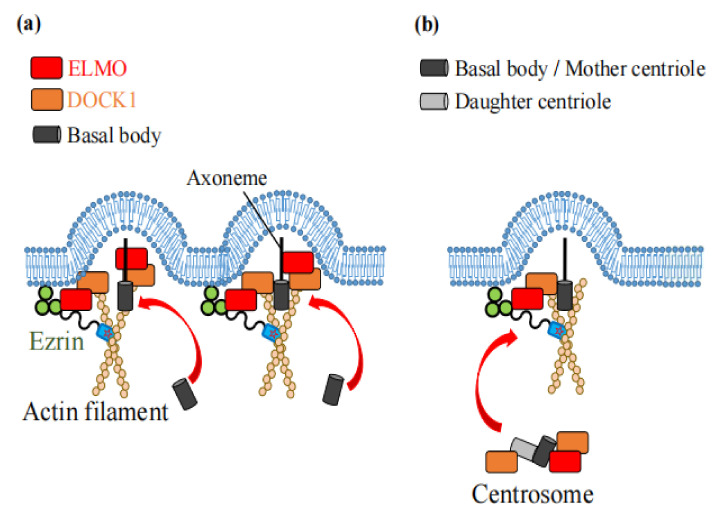
Ezrin functions as an F-actin binding protein for ciliogenesis. (**a**) In motile cilia, ezrin causes apical actin enrichment via interaction with actin filaments. Subsequently, the accumulation of BBs (shown by a black column) in the apical region promotes ciliary formation. Ezrin also interacts with ELMO (shown by a red box), which binds to DOCK1 (shown by an orange box). ELMO localizes along the axonemes and BBs in motile cilia. (**b**) When cells arrest in the G_0_ stage of the cell cycle, BB arises from the mother centriole (shown by a black column) of the centrosome, and forms the base of the primary cilium. In primary cilia, ELMO is localized at the mother centriole, whereas DOCK1 is localized to both mother and daughter centrioles.

**Figure 9:**
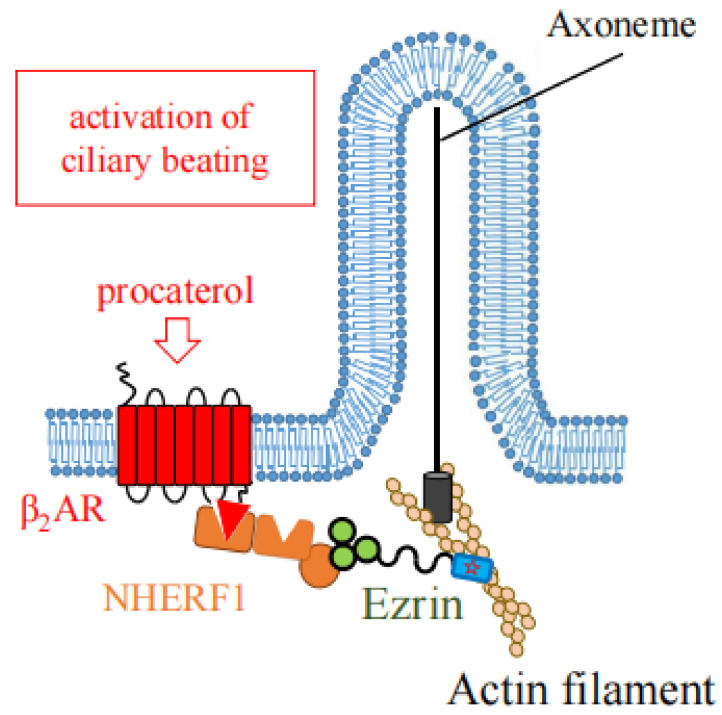
Ezrin links the β2AR to actin filaments for stable expression of the receptor at the cell surface of airway ciliary cells. At the apical surface in motile ciliary cells, multiprotein complexes consisting of β2AR-NHERF1-ezrin links to the actin cytoskeleton to efficiently activate ciliary beating via the reception of procaterol.

F-actin plays essential roles in the generation of cilia: ciliogenesis. Ezrin functions as an actin-binding protein which interacts with basal bodies (BBs) throughout the apical region, and enables the generation of motile and primary cilia (Figure 8a,b). Ezrin also plays a role in the activation of ciliary beating by cross-linking the β_2_AR to the actin cytoskeleton for its stable expression in apical membranes (Figure 9).

### 5.1. Roles in Motile Cilia

In the process of generating motile cilia, the accumulation of the actin cytoskeleton in the apical region causes the apical expansion of multiciliated cells. This structuring of the actin cytoskeleton leads to the orientation and docking of BBs to the apical membrane, and enables the generation of motile cilia [62]. Ezrin is localized to the apical region of airway ciliated cells, and is involved in the docking of BBs to the apical membrane. This apical localization of ezrin disappears in the mice lacking the Forkhead box protein J1 (Foxj1), which is a master transcriptional factor required for ciliogenesis [63]. The loss of Foxj1 caused a loss of the ability of BBs to anchor to the apical cytoskeleton due to calpain-dependent degradation of ezrin and NHERF1. This in turn led to the disappearance of axonemal formation in the apical region in mouse tracheas [64]. Similarly, in human airway epithelial cells, treatment with interleukin-13 induced the loss of ezrin from the apical region and subsequently resulted in the loss of cilia [65].

Ezrin also interacts with the ELMO protein, which binds to DOCK1. The ELMO-DOCK1 complex functions as a GEF of Rac1. In human respiratory epithelial cells, ELMO localizes along the ciliary axonemes and BBs. In *Xenopus* and zebrafish embryos, ELMO or ezrin knockdown impaired BB docking and its correct spacing in the apical membrane [31].

In mouse airway ciliary cells, a β_2_AR agonist procaterol activates ciliary beating via increasing intracellular cAMP concentration. Recently, we found that ezrin knockdown in mice leads to the suppression of ciliary beating activated with procaterol, without changes of ciliary morphology and BB orientation in the airway epithelium [25]. In this case, ezrin functions as a cross-linker between β_2_AR and the actin cytoskeleton as reported in Section 1 (Figure 9).

### 5.2. Roles in Primary Cilia

Ezrin is also important for the generation of primary cilia, and their dysfunction causes various ciliopathies. The generation of primary cilia is disturbed by the loss of binding of ezrin to F-actin in zebrafish embryos, which was found in via ezrin knockdown using a translation-blocking morpholino (TB-MO) [31]. In fact, ezrin knockdown causes prominent hydrocephalus and pronephric cyst formation. These symptoms are characterized by primary ciliary dyskinesia in zebrafish embryos. In addition, the overexpression of either a phosphorylation-deficient (T564A) or phosphorylation-mimetic mutant ezrin (T564D) caused pronephric cyst formation [31]. In pronephric tubules of zebrafish embryos, the knockdown of ELMO, DOCK1, or Rac1 increased ezrin phosphorylation, accompanied by prominent pronephric cyst formation. The overexpression of a phosphorylation-deficient ezrin (T564A) mutant prevented this pronephric cyst formation [31]. These results further suggest that ezrin is a key protein which regulates the formation of primary cilia through its phosphorylation by ELMO/DOCK1/Rac1.

Inositol polyphosphate 5-phosphatase E (INPP5E) hydrolyses the 5-phosphate of PtdIns(3,4,5)P_3_ and is co-localized with ezrin at the apical region. Its gene is one of the causative genes of Joubert Syndrome (JBTS). The mutations of this gene cause primary cilium signaling defects, ciliary instability, and ciliopathies. It is interesting that the overexpression of the ezrin gene rescued the loss of primary cilia found in INPP5E knockdown zebrafish embryos. Therefore, ezrin plays important roles in the accumulation of F-actin in the apical region via its binding to PtdIns(4,5)P_2_ [66].

## 6. The Roles of Ezrin in Cancer

High expression of ezrin is linked to poor survival in many cancers, including carcinomas of the breast [67,68], esophagus [69], tongue [70], lung (non-small-cell lung cancer) [71], stomach [72], endometrium [73], and osteosarcoma [74], rhabdomyosarcoma [75], soft tissue sarcomas [76] and melanomas [77], and brain astrocytic tumors [78] except for bladder tumors. Athanasopoulou, et al. reported that the down-regulation of ezrin in urothelial bladder tumors is associated with aggressive tumor features and invasiveness [79].

Ezrin is a key regulatory protein and necessary component in the metastasis of these tumors [67,74,75,80]. Meta-analysis suggests that ezrin is a potential prognostic marker in cancer patients [81]. Ezrin is a potential prognostic biomarker especially for the recurrence of breast cancer [82]. The ezrin corner score (ECS) which represents the expression of ezrin in poorly differentiated clusters seems to be of potential value for the prognosis of colorectal cancers [83].

One of the mechanisms in which ezrin is involved in metastasis is its role in the induction of epithelial–mesenchymal transition (EMT) [68,84]. In tongue squamous cell carcinomas (TSCCs), EGF induces EMT and metastasis through the phosphorylation of ezrin at Tyr^353^ by the EGF receptor protein-tyrosine kinase, and the activation of AKT and NF-kB [84].

In A549 lung cancer cells, a TGF-β1-induced EMT process was reported to be mediated by the interaction between ezrin and podocalyxin. Suppressing ezrin expression limited the morphological changes and actin filament remodeling, and decreased cell migration and invasion during EMT [85].

The other mechanism by which ezrin is involved in invasion and metastasis is its interaction with CD44. CD44 is one of the key components determining the tumor-endothelial interaction and tumor-stromal interactions [86]. A cluster of CD44 acts as a tumor promoter by playing a role as a platform in the effective presentation of growth factors to their receptors especially for receptor tyrosine kinases. In this process, ezrin acts as a link between CD44 and the actin cytoskeleton as reported previously. However, it should be noted that CD44 acts as a tumor suppressing factor in certain tumors such as neuroblastomas and prostate cancers [87]. It was also reported that CD44 (CD44 containing variant exon v6 sequences) forms a complex with HGF and its receptor tyrosine kinase Met, and this complex formation and the signal transfer from Met to MEK and Erk depend on the interaction between CD44 and ezrin [88].

Recently, Xue, et al. reported that ezrin has a direct role in the control of the Hippo pathway, which regulates organ size, cell fate, and carcinogenesis through the regulation of cell proliferation and apoptosis. Ezrin phosphorylation (at Thr^567^) activates a transcription factor, yes-associated protein (Yap1), which promotes gene expression involved in cell proliferation and suppresses gene expression involved in apoptosis in hepatocellular carcinomas (HCCs) [89]. Ezrin promotes pancreatic cancer cell proliferation and invasion through the interaction with and nuclear translocation of YAP. YAP and ezrin are overexpressed in pancreatic cancer, and their expression correlates with poor prognosis in pancreatic cancer patients [90]. Ezrin also plays an important role in the maintenance of the size/mechanical force and proliferation of skin fibroblasts through the interaction with YAP. Its depletion inhibits fibroblast proliferation and mechanical force via impaired YAP activity [91].

It should be noted that ezrin expression is up-regulated and correlates with HER2 expression in human HER2-positive breast cancer cell lines and invasive breast cancers from human patients. Ezrin stabilizes the multiprotein complex consisting of HER2, plasma membrane Ca^2+^-ATPase 2 (PMCA2), NHERF1, and HSP90 at the specialized membrane domain, which results in membrane retention of HER2 contributing to tumor progression [92].

## 7. The Role of Ezrin as the Regulator of mRNA Translation

Ezrin shows a novel (non-canonical) function as a regulator of mRNA translation. According to proteomics studies of the mRNA complex (mRNA interactome), ezrin was listed as one of the RNA binding proteins (RBPs) [93,94]. Therefore, ezrin seems to be a component of a messenger ribonucleoprotein. Çelik, et al. reported that ezrin directly binds with DEAD-box RNA helicase DDX3, which functions in a variety of RNA metabolism pathways. Ezrin regulates the mRNA translation process by blocking the RNA helicase activity of DDX3 [95].

## 8. Small Molecules of Ezrin Inhibitor

**Figure 10:**
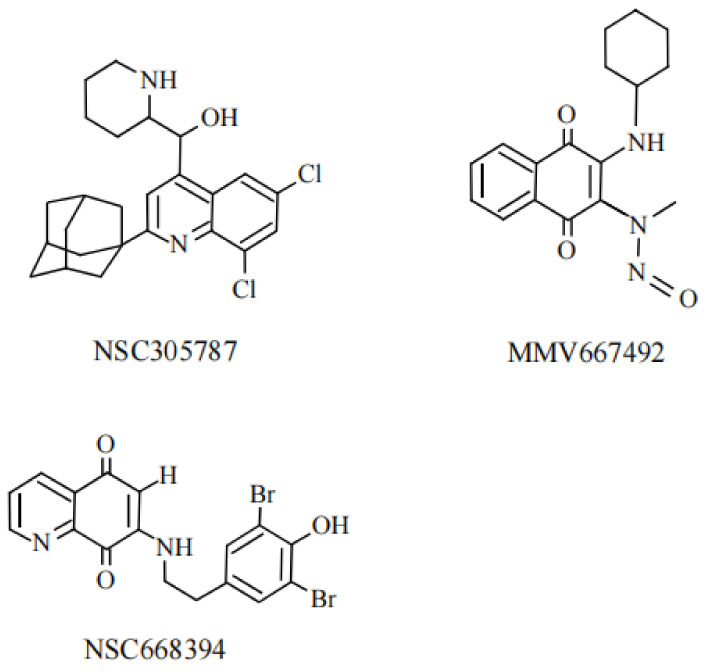
The chemical structures of ezrin inhibitors, NSC305787, NSC668394, and MMV667492.

Ezrin is a potential anti-metastatic target protein. Therefore, small molecule inhibitors of ezrin should be considered as potential tools to block metastasis [82]. NSC305787 and NSC668394 (Figure 10) are small molecule inhibitors which directly bind to ERM proteins [96]. They inhibit ezrin phosphorylation at Thr^567^ which results in decreased expression of the Rho pathway and reduced filopodia formation in ectopic endometrial stromal cells [97]. NSC305787 also inhibited the pulmonary metastasis of osteosarcomas [98]. The systemic inhibition of ezrin by NSC668394 suppresses the migration and metastasis of breast cancer cells at the distal axillary lymph node and lungs [99]. Çelik, et al. reported that MMV667492 (Figure 9), which is a quinolone-based derivative, exhibited more potent anti-ezrin activity including inhibition of metastasis of osteosarcoma compared to NSC305787 [100].

## 9. Conclusions

As introduced in the previous sections, ezrin is a multi-functional protein associated with cortical actin filaments, which is involved in cell migration, endocytosis, survival, signal transduction, and translational regulation, and so on. Its function is strictly regulated by many signal molecules through phosphocycling and dynamic conformational changes. In fact, its up-regulation through overexpression and/or hyper-activation is linked to poor prognosis in many kinds of tumors. Therefore, ezrin has been attracting attention not only from the viewpoints of basic sciences but also from the clinical points of view. Future studies are awaited.

**Table 1:** Ezrin-binding proteins and their functional roles.

Proteins	Functional Roles (Cell/Tissue)
Direct Interactions:	
Membrane Proteins:	
CD44	Cell surface adhesion receptor involved in cell-cell and cell-matrix interactions [10].
CD43	Sialoglycoprotein involved in T cell activation (in leukocyte) [11].
ICAM-2	Ig-like cell adhesion molecule (in leukocyte and endothelial cell) [11].
ICAM-1	Ig-like cell adhesion molecule (in leukocyte and endothelial cell) [12].
L-selectin	Cell adhesion molecule which induces leukocyte transendothelial migration (in leukocyte) [14].
NHE1	Transporter exchanging protons for sodium ions (in fibroblast and renal tubule) [16].
P-gp	Multidrug resistance (in intestine, renal tubule and hepatocyte) [17].
ATP11C-b	Phospholipid flippase (in leukocyte) [19].
NHERF1 (EBP50)	Scaffold protein: apical localization of membrane protein [20].
NHERF2	Scaffold protein: apical localization of membrane protein [21].
Podocalyxin	Sialoglycoprotein which forms the glomerular filtration slits (podocyte) [37].
CD95	Death receptor (in leukocyte) [50].
**Rho-related proteins:**	
PLEKHG6	Scaffold protein: apical localization of membrane protein [30].
RhoG	Formation of microvilli and membrane ruffles [30].
ELMO	Ciliary basal body migration [31].
ACAP4	Localization of the gastric proton pump for gastric acid secretion [32,33].
Rho GDI	Reorganization of actin filaments [36].
**AKAP:**	
PKA	cAMP-dependent protein kinase; parietal cell activation, CFTR-activation [55,56].
**Indirect interactions:**	
**Membrane proteins:**	
CFTR	Chloride channel (in lung, intestine and renal tubule) [22].
NHE3	Transporter exchanging protons for sodium ions (in gastrointestinal tract and renal tubule [23].
Npt2a	Sodium-dependent phosphate transporter (in renal tubule) [24].
β_2_AR	Adrenergic receptor (in airway) [25].

## Figures and Tables

**Figure 1:**
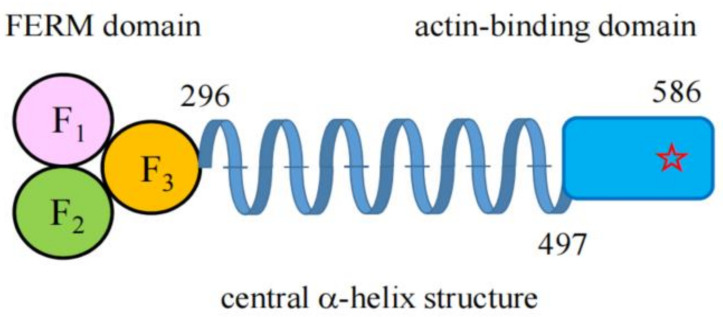
Schematic structure of ERM proteins. ERM proteins are divided into three parts; that is, the N-terminal FERM domain (296 amino acid residues), the central α-helix structure, and the C-terminal F-actin binding domain shown in a blue box (90 amino acid residues). The FERM domain consists of three lobes (F1, F2, and F3) which form a compact clover-shaped structure. An asterisk in the C-terminal F-actin binding domain represents a functionally important Thr residue (Thr^567^) which is phosphorylated by several protein kinases. Amino acid numbers shown here are based on those of mouse ezrin. The overall structure is conserved in all ERM proteins.

**Figure 2:**
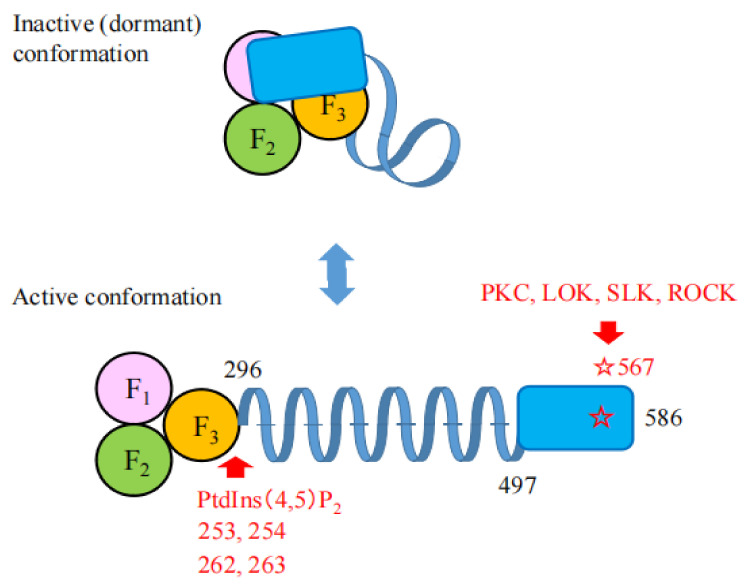
The function of ezrin is strictly regulated by the interaction between the N- and C-terminal domains. The upper figure represents the inactive (or dormant) form of ezrin. In this form, the N-terminal FERM domain interacts with the C-terminal domain, which makes ezrin inactive and dormant. In the lower figure, the binding of PtdIns(4,5)P_2_ to the F3 lobe of the FERM domain (amino acid numbers 253, 254, 262, and 263 shown by an upward red arrow) and the subsequent phosphorylation of Thr^567^ residues (shown by a red asterisk) on the C-terminal domain by PKC, LOK, SLK, or ROCK (shown by a downward red arrow) open the inactive form into the active form. The activated ezrin is recruited to the apical membrane. These processes of activation seem to be common among the ERM proteins.

## Data Availability

Not applicable.
